# Dietary Practices and Nutrient Intake of Internally Displaced School Children in the West Region of Cameroon

**DOI:** 10.1155/2023/9954118

**Published:** 2023-02-17

**Authors:** Nwachan Mirabelle Boh, Ejoh Richard Aba, Chefu Burnice Lemfor

**Affiliations:** University of Bamenda, Department of Nutrition, Food and Bioresource Technology, Bafoussam, Cameroon

## Abstract

**Background and Aims:**

Poor diets and subsequent malnutrition are among the greatest current societal challenges triggering immense health and economic burden especially among populations that are forcibly displaced. It is indispensable to establish the dietary patterns of any population, especially in displaced populations, in order to develop and effectively implement interventions for the specific population.

**Methods:**

A cross-sectional study was conducted to assess dietary practices and nutrient adequacy of 307 internally displaced pupils aged between 5 and 15 years in the West Region of Cameroon. Pretested, structured interviewer questionnaires were used to collect data on the demographic and socioeconomic status of the children and their caregivers, the nutrition knowledge of caregivers, the dietary practices of the children, dietary diversity, their food sources, and coping strategies that were used during food shortage. SPSS version 23 was used to analyze the data. The dietary diversity of the children was assessed at the individual level using FAO method of assessing women's dietary diversity score.

**Results:**

Out of the total 307 children, 148 (48.2%) were boys and 159 (51.8%) were girls. During food shortages, most of the mothers/caregivers (72%) used borrowing as the main auxiliary food source and others (28%) used food as payment for work or begging. A majority of the children (56%) usually ate only two times in a day. Most of the children were of unacceptable or low dietary diversity (66.2%). The most frequently consumed food group was cereals as it was eaten by 21% of the children, seven or more times per week; meanwhile, the least was animal products with only 3% of the children consuming it seven or more times weekly. Their diets were energy-deficient (1640.5 ± 1.64 kcal) and unbalanced with daily inadequate protein (18.45 ± 1.13 g), vitamin A (470.27 ± 1.38 *μ*g), and iron (4.02 ± 0.08 mg) intake. The nutrition knowledge of the mothers/caregivers was poor as less than half (41%) of them had an acceptable nutrition knowledge.

**Conclusion:**

The high prevalence of poor dietary patterns and poor nutrition knowledge imposes the necessity of developing nutritional interventions and education strategies aimed at promoting healthy eating habits in the children.

## 1. Background

Dietary intake as a pivotal element contributing to human health and well-being is of great significance, and its role on nutritional status especially during childhood is more renowned and of great concern [1]. Nutritional intake during childhood has a distinctive direct effect on the formation of long-term eating habits in children [1]. Dietary practices are significant precursors of disease and good health. In comparison to individual foods or nutrients, dietary patterns are more accurate in describing the relation of diet to health and disease since neither one nutrient nor one food effectively describes dietary behavior [2]. Healthy eating habits result in a stronger immune system and less morbidity, prevent noncommunicable diseases, and improve health. Appropriate and healthy nutrition is a key to a better quality of life. Epidemiological studies have shown that changes in lifestyle during recent years, especially changes in nutritional habits, diet, types of food, and cooking time, may be responsible for the increasing rates of noncommunicable diseases [3–5]. Dietary intake patterns and undernutrition are associated with different immediate complications and major life-long consequences including functional impairment in adult life, reduced work capacity, and decreased economic productivity of the individual [6]. Poor dietary pattern and undernutrition deprive children of fundamental vitamins and minerals, render them more susceptible to infections and chronic diseases, and are responsible for 3.1 million child deaths annually [7]. Consequently, quality and quantity of children's diet have become the major concerns for researchers. This is because most children do not meet the recommended standards of dietary guidelines and are devoid of healthy dietary habits [7]. Poor diets and subsequent malnutrition are among the greatest current societal challenges, triggering immense health and economic and environmental burdens especially among displaced populations [8]. Poor dietary practices are highly prevalent among children in Cameroon as only 20% of them attain a minimum dietary diversity, and about 43% of them attain the minimum meal frequency [8]. According to UNICEF [9], most children in Cameroon are malnourished not because of the lack of food but because of the poor dietary practices. The prevalence of severe acute malnutrition among children in Cameroon is 2.72% [9].

Among the factors that affect the dietary intake of the children such as income, food availability, and culture, nutrition knowledge of the mothers has been proven to be one of the most important. Adequate knowledge and skills to eat a variety of foods in the correct quantities and combinations is a prerequisite to healthy dietary practices and achieving good nutritional status [10]. Lack of nutrition information and knowledge, which is accompanied by undesirable dietary habits and nutrition-related practices, attitudes, perceptions, and sociocultural influences, further downgrades dietary quality [11, 12]. Therefore, access to information on the level of nutrition knowledge of the caregivers of these children will identify nutrition education priorities that can be used to improve their nutrition knowledge and hence dietary practices.

Although the school age is known as a critical stage for shaping eating and lifestyle habits that will last later in life and have an effect during adulthood and even old age [13], the diet of school-aged children in the middle- and low-income countries is usually poor in fruits, vegetables, and animal products, leading to inadequate intake of iron, vitamin A, and zinc [3]. Augmenting cognitive and physical development in primary school children through appropriate dietary practices could have life-long benefits [13]. School-aged children in developing countries are especially vulnerable to inadequate consumption of micronutrient-rich foods, dietary taboos, lack of access to health care, and inefficient utilization of available micronutrients caused by food insecurity, infections, and parasitic infestations among other reasons [14]. These causes are more challenging when children are displaced during emergencies [15].

Conflict will unavoidably cause loss of lives, physical injuries, widespread mental distress, an aggravation of existent malnutrition (particularly among children), and outbreaks of communicable diseases, especially among internally displaced and refugee populations [16]. It also lessens people's personal security and restricts their access to food, clean water, sanitation, shelter, and health services. Of the forced migrants, internally displaced persons (IDPs) are among the most vulnerable [17].

Although the local government and international health organizations have in recent years implemented a variety of interventions to promote healthy eating behaviors of children, they have had limited impact. This might be attributed to insufficient understanding of their dietary habits at different ages and the necessary interventions that should be implemented in accordance with different age groups [18]. Shepherd and Egham [18] revealed that dietary influences vary with age, and not all interventions are suitable for all age groups. Yet, to date, very limited research has examined nutritional practices of pupils in Cameroon. Hence, access to high-quality data on the existing practices of this age group and maternal knowledge relating to nutrition is crucial as it would assist in prioritizing and setting up cautious, evidence-based nutrition intervention programs, targeting the nutritional problems that are of substantial concern [14]. The purpose of the study was therefore to assess the dietary patterns of internally displaced school children in the West Region of Cameroon and the nutrition knowledge of their caregivers through a participatory baseline survey.

## 2. Methodology

### 2.1. Study Site

The West Region, whose headquarter is in Bafoussam, is located in the central-western portion of the Republic of Cameroon. It has a surface area of 14,000 km^2^ and is located in the central-western part of the Republic of Cameroon. It is divided into eight divisions. The West Region borders the Northwest Region to the northwest, the Adamawa Region to the northeast, the Centre Region to the southeast, the Littoral Region to the southwest, and the Southwest Region to the west. Among Cameroon's ten regions, the West Region is the smallest region in terms of surface area, but it has the highest population density. The West Region is one of the regions with the greatest number of IDPs in the country [19].

### 2.2. Study Design and Sample Size Estimation

A cross-sectional study was carried out in the West Region of Cameroon in September 2021. The sample size was calculated using Fischer's formula [20].

(1) $$\begin{array}{c} n=\frac{Z^{2}xP\left(1-P\right)}{e^{2}}, \end{array}$$

where *n* is the desired sample size; *Z* is the standard normal deviation at confidence level of 95%, set at 1.96; *p* is the proportion of the target population estimated to have the characteristics being measured. The proportion of children with minimum dietary diversity in Cameroon (*P*) is 20% [8]; and *e* is the margin of error which is 5% or 0.05.

The calculated sample size was 246 children. A nonresponse rate of 10% was considered, and the sample size was increased to 307. The study participants were pairs of caregivers and their children.

### 2.3. Inclusion and Exclusion Criteria

Study participants included internally displaced school children in the age of 5-15 years and are enrolled in primary schools located in the West Region of Cameroon. Internally displaced children who were sick were excluded from the study.

### 2.4. Data Collection

Data was collected using a pretested structured interviewer questionnaire in September 2021. The questions were answered by the caregivers of the children. The questionnaire was used to collect information on demography, socioeconomic characteristics, drinking water sources, the region where the child lived before the crisis, consumption of food supplement, the source of foods/food assistance, the year of child's displacement, coping strategies to food insecurity, dietary practices of the children, and their caregiver's knowledge. The questionnaire used for the study is available from the corresponding author upon request.

#### 2.4.1. Sampling Procedure

The West Region was purposely chosen because this region is one of the regions with the greatest number of IDPs in the country. The Menoua, Bamboutos, and Mifi divisions in the West Region were also purposely chosen because these divisions host the greatest number of IDPs in the region. With the aid of the Divisional Delegation of Basic Education, the names of schools with an English-speaking subsystem of education in the respective divisions with their population of the displaced school-aged children were noted. A random selection was made from a list of 22 schools which had large numbers of displaced children. A total of 10 schools were selected for the survey. The multistage random sampling method was then used to ensure that the number of children taken from each division was proportional to the number of displaced children in the division. Random sampling of boys and girls was done to obtain the required sample for the study.

#### 2.4.2. Assessment of Dietary Practices and Children's Dietary Intake

*(1) Nutrient Intake Assessment*. Dietary intake and adequacy of energy, protein, vitamin A, and iron were assessed using a previously validated 24-hour recall. The caregivers were asked to recall all foods and beverages consumed by their children during the past 24 hours according to principles described by FAO [2]. The daily nutrient intake in terms of energy, protein, vitamin A, and iron was calculated with the help of the West African Food Composition Table [21]. The nutrient values were compared to the recommended dietary intake (RDI) for school-aged children [22].

*(2) Dietary Diversity*. Diet diversity of the children and their micronutrient adequacy were assessed at the individual level using the method described by Liu et al. [23] and Oumer and Berhanu Abebaw [24]. According to previous studies, this method which is based on 10 food groups outperforms indicators based on other numbers of food groups in children [25, 26]. The 10 food groups are (1) cereals, white roots and tubers, and plantains; (2) pulses (beans, peas, and lentils); (3) nuts and seeds; (4) dairy; (5) meat, poultry, and fish; (6) eggs; (7) dark green leafy vegetables; (8) other vitamin A-rich fruits and vegetables; (9) other vegetables; and (10) other fruits. To exclude very small quantities, the scoring criteria provides 1 point (score) for each food group when intake is equal to 15 g. If not, the point is 0 [27]. The total number of food groups consumed by an individual constitutes the individual's dietary diversity score. A cut-off point of 5 was used to define the minimum dietary diversity (MDD); i.e., the children with dietary diversity score (DDS) 5 or more were defined as meeting MDD, and those with DDS 4 or less were considered as not meeting MDD as recommended by the FAO [2]. An individual who consumed 5 food groups is of medium dietary diversity, and from 6 and above was referred to as high diversity score [2].

*(3) Frequency in Food Selection*. The food frequency questionnaire was used to collect data on the number of times the children consumed each food item over the last seven days prior to the study. The questionnaire was adopted from FAO [2] and modified to include a list of 59 locally available foods which were identified in the local markets and households. The questionnaire consisted of two components, namely, a list of the food items and a set of frequency-of-use response categories. This includes how often each of the foods included in the food frequency questionnaire was eaten in a week. Frequency scores were assigned for each food depending on how often a food is consumed (seven or more times, 5-6 times, 3-4 times, 1-2 times, and 0 = never).

#### 2.4.3. Caregiver's Knowledge

The level of nutrition knowledge of the mothers/caregivers was assessed using a questionnaire made up of 10 multiple-choice questions and marked on 10.

### 2.5. Data Analysis

Data were entered in Microsoft Excel and then transferred to IBM Statistical Packages for Social Sciences (SPSS version 23) for coding and analysis. A cut-off of 75% of the RDI for the nutrients was used to indicate adequacy or inadequacy. Food frequency was assessed based on the number of times each food item was eaten in a week. The total number of food groups consumed by an individual constituted the individual's dietary diversity score. When the dietary diversity score was less than four, the individual is said to be of low dietary diversity. An individual who consumed 4 or 5 food groups is of medium dietary diversity, and from 6 and above was referred to as high diversity score [2]. Each item on the caregiver's nutrition knowledge was scored as 1 for a correct response and 0 for an incorrect response. Those who scored below 3 out of 10 were considered to have very poor nutrition knowledge. Those who scored 3 or 4 out of 10 were considered to have poor nutrition knowledge. Those who scored 5 or 6 out of 10 were considered to have averagely good nutrition knowledge, and those who scored 7 or 8 out of 10 were considered to have very good nutrition knowledge while those who scored 9 or 10 out of 10 were considered to have excellent nutrition knowledge. Descriptive statistics such as frequency mean and standard deviation were calculated and presented in tables and graphs. The results were expressed as means and standard deviations, frequencies, and percentages. Significant differences were set at *P* value < 0.05.

## 3. Results

### 3.1. Demographic Characteristics of the Children

Out of the 307 children who participated in the study, 51.8% of the children were female, whereas 48.2% were males as shown Table 1. Most of the children (68.4%) were of age 10-15 years while the age range of 5-9 years comprised of 40.7% of the children. The mean ± SD age of the study population was 10.3 ± 2.48, with an age range between 5 and 15 years. Most of the displaced children (68.4%) in the West Region were from the Northwest Region while 31.6% of the children were from the Southwest Region. The frequency table below reveals that an equal percentage of children (28.7%) was displaced in 2018 and 2019. A slightly lower percentage (26%) of the children was displaced in 2017. The percentage of displaced children dropped in 2020 (12%) and continued dropping (4.5%) in 2021.

### 3.2. Demographic Characteristics and Socioeconomic Status of the Mothers/Caregivers

From Table 2, it was noticed that mothers had varied levels of education ranging from no formal education to university level. Regarding mothers' educational status, some of them (2.9%) had no formal schooling, 34.9% had primary school as their highest level of education, slightly more than half of the mothers (52.4%) ended with secondary education level, and only 9.8% attended higher education. Unemployment was high among the mothers/caregivers as almost half of them were unemployed (49.2%). About 42.7% of the mothers/caregivers were self-employed; meanwhile, the least proportion (8.1%) of the mothers had paid jobs. It was revealed that matrimonial status varies greatly among mothers/caregivers of the displaced children. The proportion of mothers who were married is 65.8%, 22.1% of them had never married, 6.5% were widows, and 5.5% were divorced. The household income of the families of these displaced children is very poor as most of the families (76%) had a monthly income below 50.000 francs, whereas the rest (21.2%) had an income ranging from 50.000 to 150.000 francs. A few families (3.6%) had a family income above 300.000 francs as shown Table 2. The households where these children live were crowded as 52.4% of the families had a household size between five and eight family members; meanwhile, 35.9% of the households were overcrowded with more than eight people. Only 13.7% of the households were not crowded as it had less than five members. These revelations imply that due to the large household sizes coupled with low-income levels, food intake was likely to be negatively affected. Most of the displaced children (55.7%) are living with their mothers. Others (39.7%) are living with a family relative. A few of them are with family friends (4%). The table also reveals that most of the mothers/caregivers (57.0%) are in the age group of 25-50 years, whereas 34.9% of them were aged below 25. Only 8.1% of the mothers/caregivers were above 40 years. The table below also reveals that 26.4% of the mothers were living with their children under another family, and 69% of them were renting, while 4.6% of the mothers/caregivers were living in their own houses. Regarding the source of drinking water for the household, this study revealed that a small proportion of them drink water from the well, river, and stream which are known as bad water sources; meanwhile, most of the families (76.2%) had good sources of drinking water, which include tap water, spring, and bottled water. The pit toilet is the most common type of toilet used by the families of these displaced children and is used by 80.8% of them. A small proportion of them (16.3%) are using the flushing toilet, and some of them (2.9%) do not have a toilet. Most of the respondents (69.4%) were Christians by religion, while 23.5% were Muslims and the rest (2.3%) were pagans.

### 3.3. Dietary Sources

#### 3.3.1. Food Assistance from any NGO within the Past One Month

**Figure 1:**
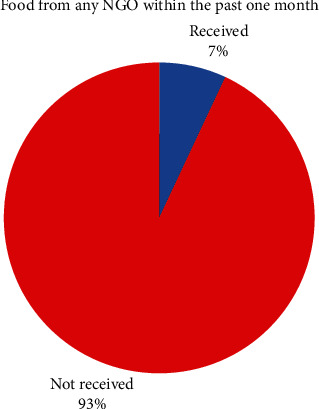
Food from any NGO within the past one month.

Most of the mothers/caregivers (93%) said that they had not received any food assistance from a nongovernmental organization (NGO) within the past one month as shown Figure 1. Only 7% of them acknowledged that they had received any food assistance from an NGO within the past one month prior to the study.

#### 3.3.2. Main Auxiliary Food Sources

**Figure 2:**
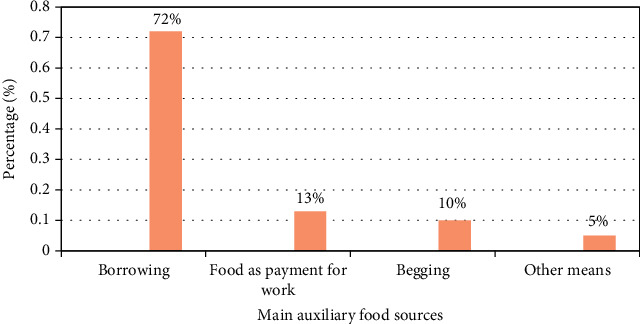
Main auxiliary food sources.

Figure 2 reveals that 72% of the mothers/caregivers used borrowing foods as their main alternative means of having food during food shortages, 13% of the families used food as payment for work, 10% of the families used begging as their main auxiliary food source, and 5% of the families used other means such as trade by barter and selling of household goods.

### 3.4. Dietary Practices

#### 3.4.1. Meal Frequency per Day

**Figure 3:**
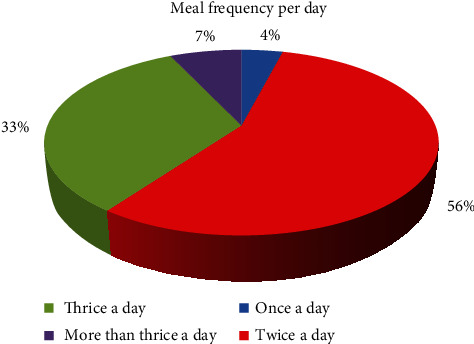
Number of eating times in a day.

Figure 3 shows that majority of children (56%) usually ate only two times in a day which is below their acceptable daily minimum meal frequency (three or four times per day). This is probably because their caregivers lack resources to afford three meals in a day for the children, considering the fact most of the families have a very low income (less than 50.000 francs CFA). Far less than half of the children (40%) met their daily minimum meal frequency as they were eating three (33%) or more than three times (7%) in a day. The least percentage was made up of those who ate only once in a day (Figure 3).

#### 3.4.2. Consumption of Food Supplement

Nearly all of the children (99.7%) were not receiving any food supplement. Only a negligible number of the mothers (1.3%) affirmed that their children were receiving any food supplement.

#### 3.4.3. Food Group Frequency

Table 3 reveals that the diet of the displaced children was monotonous comprising mainly of cereal-legume meal with up to 97.7% and 78.2% of the children, respectively, consuming cereals and legumes weekly. The percentage of pupils who ate roots and tubers weekly was 60.4%; fats, oil, and sugars was 100%; and vegetables was 64.0%. Apart from fats, oil, and sugars, cereals were the most frequently consumed food group with up to 34.2% of the children consuming it 5-6 times in a week and 21% of them eating it more than seven times per week.

Animal products which constitute dairy, fish, meat, eggs, and chicken were the least frequently consumed food group as only 3% of the children consumed it more than seven times in a week. The weekly frequency of consumption of fruits and vegetables was low overall as up to 57.3% and 36% of the children, respectively, never ate these food groups within the period of seven days.

#### 3.4.4. Dietary Diversity

*(1) Proportion of Food Groups Consumed by the Study Respondents*. Considering the different foods consumed 24 hours prior to study (Table 4), nearly all the children (99.3%) ate starchy staples, and almost three-quarters (73.0%) consumed other vegetables (green beans, tomato, onion, eggplant, cucumber, okro, and other locally available vegetables). Consumption of protein-rich foods among the children was poor as only 31.3%, 25.0%, 29.7%, and 9.1% of them ate beans and peas; nuts and seeds; meat, poultry, and fish; and eggs, respectively. Also, only a few children consumed vitamin A-rich foods (eggs = 9.1%, dairy products = 12.7, vitamin A-rich vegetables and fruits = 19.5%, and dark green leafy vegetables = 32.3%). Daily intake of iron-rich foods (dark green leafy vegetables (32.3%), eggs (9.1%), and meat, poultry, and fish (29.7%)) was minimal (Table 5). The most commonly consumed food group was cereals, tubers, white roots, and plantains which were eaten by 99.3% of the children. The least consumed food group among the 10 food groups was the group of eggs as it was eaten by only 6.8% of the children.

**Figure 4:**
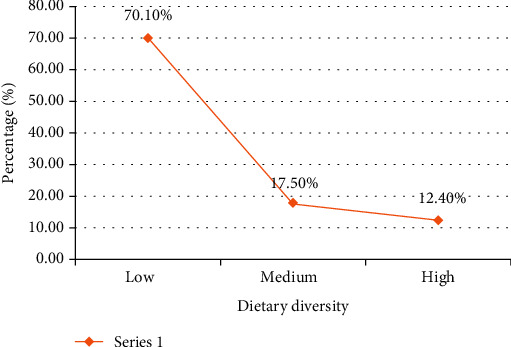
Dietary diversity scores of the children.

*(2) Dietary Diversity among the Children*. From Figure 4, it was found that most 66.2% of the pupils were of low or unacceptable dietary diversity. The mean (± standard deviation) individual dietary diversity score (IDDS) of the study children was 3.43 ± 1.02 (score range: 1–10). The frequency of participants with an acceptable dietary diversity score was only 104 with a percentage of 33.8%. Among the 33.8% who were able to meet their minimum acceptable dietary diversity, which is a proxy indicator for micronutrient adequacy, 21.4% of them had a medium dietary diversity score (score of 5 or 6) while 12.4% of them had a high dietary diversity score (7-10).

#### 3.4.5. Nutrient Intake of the Children

The mean daily nutrient intake of the children is presented in Table 5. It was observed that the mean daily intake of all the nutrients was inadequate as it was below the RDI with that of iron being the furthest away from the RDI. The mean daily intake of energy, protein, vitamin A, and iron in the children was 1640.5 ± 1.64 kcal, 18.45 ± 1.13 g, 470.27 ± 1.38 *μ*g, and 4.02 ± 0.08 mg, respectively.

Calorie consumption was better compared to consumption of protein among these children. Most of the children (61.2%) were able to meet their RDI for energy whereas only about half of them (50.5%) were able to meet the RDI for protein.

### 3.5. Nutrition Knowledge of the Mothers/Caregivers

#### 3.5.1. Level of Nutrition Knowledge on Various Aspects of Nutrition

As shown in Table 6, most of the mothers/caregivers (72.9%) understood what comprised a balanced diet and 65.1% were aware of how to achieve a good nutritional status. The most common knowledge among the mothers was knowledge on good cooking practices as 96.1% of the mothers/caregivers got it right. Other areas where good knowledge was observed among most of the mothers/caregivers include awareness of what comprised a balanced diet (72.9%) and knowledge of how to achieve good nutritional status (65.1%). It was noticed that not up to half of them had knowledge in most of the domains tested. The least known domain by most of them (5.2%) was on the symptoms of marasmus. This was followed by awareness of the consequences of iron deficiency as only 8.8% of the mothers/caregivers had knowledge on that.

#### 3.5.2. Nutrition Knowledge Score

**Figure 5:**
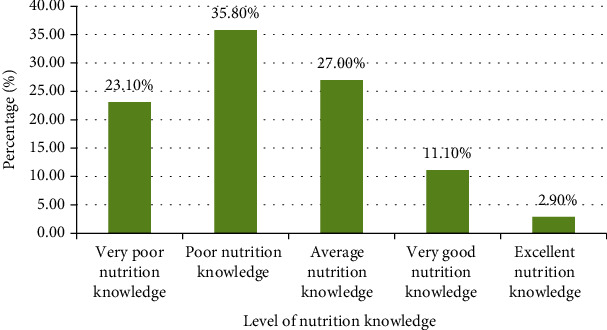
Nutrition knowledge score of mothers/caregivers of the children.

From Figure 5, the frequency of respondents who had very poor nutrition knowledge was 71, with a percentage of 23.1%, those who had poor nutrition knowledge were 35.8%, those with an average nutrition knowledge were 27%, those who had very good nutrition knowledge were 11.1%, and those with an excellent nutrition knowledge had a frequency of 9 and a percentage of 2.9%. With respect to the overall performance, the percentage of mothers/caregivers who had an acceptable nutrition knowledge (having ≥5/10) was 41% with a mean mark of 4.1 ± 1.6 on 10 which is unsatisfactory.

## 4. Discussion

The study indicated that most of the children were females (51.8%). This is in line with the report of the Ministry of Public Health and WHO [28] which recorded that there are more females in Cameroon than males. The children lived in crowded houses as 52.4% of the families had a household size between five and eight; meanwhile, 35.9% of the households were overcrowded with more than eight people. These revelations imply that due to the large household sizes coupled with low-income levels, food intake (through limited accessibility and affordability) was likely to be negatively affected. The educational status of the mothers in this study was poor as some of them (2.9%) had no formal schooling and 34.9% had primary school as their highest level of education. These findings are an indicator of a population that may face difficulties of assessing relevant information from published sources. This may perhaps imply that the caregivers may be facing challenges of assessing adequate information on nutrition knowledge.

Only 7% of them acknowledged that they had received any food from an NGO within the past one month prior to the study. This implies that although the Cameroon government and other NGOs used to distribute food to IDPs, food distribution to IDPs was not a common practice during the previous month to this study. Therefore, there were probably food shortages among the children since internally displaced people and host communities are highly dependent on integrated food and nutrition assistance [29].

This study revealed that most (72%) of the mothers/caregivers used borrowing as their main alternative means of having food during food shortages. It is a well-known fact that displaced populations always face food insecurity and therefore use auxiliary food sources as coping strategies [30]. Like the IDPs in the West Region, one of the most common main auxiliary food sources of food insecure households in the conflict areas of South Sudan was borrowing food from a friend or relative [31].

The percentage of children who met their daily minimum meal frequency in this study (40%) is slightly higher than (37.7%) which was recently reported among internally displaced children in Amhara region, Northwest Ethiopia [32]. On the contrary, it was realized that most of the school-aged children in Isfahan province, Iran, showed acceptable status for frequency of meal consumption, eating at least three times in a day [33].

In this study, it was observed the diet of the displaced children was monotonous comprising mainly of cereal-legume meal with up to 97.7% and 78.2% of the children, respectively, consuming cereals and legumes weekly. Also, the food groups that were more frequently consumed among internally displaced Somalian children were the cereals and tubers and the dairy (88.4% and 81.5%, respectively) followed by the legume and nut food groups consumed by 23% of the children [34]. Actually, monotonous nature of eating cereals is quite common for most low- and medium-income countries [8]. This may be because cereals are the most affordable and most available food group, and since most of the parents/caregivers have a very low family income, it is cheaper to purchase cereals.

The weekly frequency of consumption of fruits and vegetables was low overall as up to 57.3% and 36% of the children, respectively, never ate these food groups within the period of seven days. Animal products such as dairy, fish, meat, eggs, and chicken were the least frequently consumed food group as only 3% of the children consumed it more than seven times in a week. This may be because animal products are very expensive to purchase in Cameroon, and since these people are displaced and lack resources, they chose to forgo animal products for other less expensive food items. This can be compared to findings carried out by Fiorentino [35] and Naeeni et al. [33] among school-aged children in Cambodia and Iran, respectively, where the frequency of consumption of cereals/roots/tubers was high among the children whereas the frequency of intake of protein-rich foods was low. Furthermore, low frequency of consumption of animal products, fruits, and vegetables, which are good sources of protein, vitamin A, and iron, was reported among internally displaced children in Dream City, Iraq, and Somalia [34, 36]. The low weekly frequency of consumption of nutritious foods such as animal products, fruits, and vegetables observed in this study is probably because the consumption of these food crops in Cameroon is limited by perishability and seasonality of these food crops, poor marketing system, and inadequate processing technologies to preserve and conserve food products [37]. The agricultural production system in Cameroon is underdeveloped and requires reorientation in order to increase the availability of nutritious foods and to increase the competitiveness of Cameroonian production for international trade. Multispectral actions are hence required across food and health systems to sustainably improve access to nutritious diets in Cameroon. This can be achieved by education sector which can deliver nutritious school meals and provide nutrition education. The social protection sector can ensure that the most vulnerable such as forcibly displaced children has access to nutritious diets [37]. Again, the cultures of almost all Cameroonian communities prohibit children from consuming some nutritive delicacies like the gizzard of fowls and heart/liver of some animals [38].

Among the 10 food groups, the least consumed food group was the group of eggs as it was eaten by only 6.8% of the children. These results complement a study carried out in Somalia which revealed very low consumption of eggs among internally displaced children [34].

It was noticed that most (70.1%) of the pupils were of low or unacceptable dietary diversity. This indicates the prevailing poor micronutrient status of internally displaced school children in the West Region of Cameroon and the need to improve the intake of wholesome nutritious food through nutrition education, in addition to supplementary food. Also, poor dietary diversity seems to be a general trend in developing countries as it was also reported among school children and adolescent in most developing countries [3]. Ochola and Masibo revealed that the dietary intake of school children and adolescent in most developing countries was limited in diversity, mainly comprising plant-based food sources but with limited intake of fruits and vegetables [3]. Worst results were recorded among internally displaced children in Dusamareb, Somalia, where only 1% of the surveyed children were able to attain the minimum dietary diversity score [34]. These extremely low dietary diversity scores were probably due to the cultural practices of the participant's parents, as they were mainly comprised of pastoralists feeding children mainly with milk and cereals and also because of the presence of Al-Shabab in the area which limits any kind of humanitarian assistance or intervention [34]. The mean dietary diversity score of the children in this study (3.43 ± 1.02) was found to be lower than the mean dietary diversity score (2.4) that was reported among internally displaced children in Somalia [34].

It was observed that the mean daily intake of all the nutrients was inadequate as it was below the RDI with that of iron being the furthest away from the RDI. The extremely poor iron intake revealed by this study is similar to what was reported among Senegal and Cambodia school children where less than half (46%) of them were able to meet their RDI for iron [35]. Due to high consumption of non-hem-rich foods and foods that inhibit the absorption of iron such as phytate and oxalate and low meat intake among the study children, the absorption of iron in the present population is probably low, and therefore, prevalence of insufficient iron intake based on RDI may be underestimated. Vitamin A intake is highly dependent on seasons [39]. In Cameroon, mangos are only available from February to August, while the present study was conducted in September to December. A high proportion of children with insufficient vitamin A intake were also reported outside the mango season in a study conducted in Senegal and Cambodia school children [35]. Because most of the children surveyed had insufficient intake of vitamin A, they are at an increased risk of eye disorders, morbidity, and ceased growth since adequate vitamin A intake is important for maintaining eye health, improving growth, and reducing the risk of morbidity and mortality [40, 41]. The protein consumed by the children was mainly from plant sources which jeopardize the interpretation of these findings in terms of quality of proteins consumed. This is because animal-source proteins such as meat, fish, and eggs were consumed at a very low rate and these are good quality proteins due to the presence of essential amino acids that are easily digestible and bioavailable in the body [42]. However, animal-source proteins are expensive in terms of costs and not affordable especially for people with a low income like the case of the present study. Hence, most of them do not afford its regular consumption, and others have omitted them completely from their menu.

Unlike the internally displaced children in the West Region, Naeeni et al. [33] noticed that the daily energy and protein intakes of school-aged children in Isfahan province, Iran, were appropriate. The school-aged children in Isfahan province, Iran, were not displaced. This discrepancy further demonstrates the effect displacement on the nutrient intake of the children and hence their nutritional status.

With respect to the overall performance, the percentage of mothers/caregivers who had an acceptable nutrition knowledge (having ≥5/10) was 41% with a mean mark of 4.1 ± 1.6 on 10 which is unsatisfactory. Comparable data was reported in Ethiopia where less than half (41%) of the mothers had good nutrition knowledge on the meaning of food [43]. Conversely, Mugyia et al. [44] and Naeeni et al. [33] reported a satisfactory level of nutritional knowledge of mothers at the Etoug-Ebe Baptist Hospital Yaounde, Cameroon, and among participants in Isfahan province, Iran, respectively. The low level of nutrition knowledge demonstrated in this study may be due to low educational level of the respondents as almost 3% of the mothers had never attended a formal school and 34.9% of them had primary education as their highest level of education, contrary to the study in Yaounde where more than three-quarters (77%) of them had at least secondary education [44].

### 4.1. Strengths of the Study

The large sample size used in this study reduces the margin of error.

The results of this study are reliable as the confidence level is 95%.

### 4.2. Limitations of the Study

A single day 24-hour recall was used to interpret the inadequacy of nutrient intake.

## 5. Conclusions and Recommendations

A majority of children usually ate only two times in a day. Most of the pupils were of unacceptable or low dietary diversity. This implies micronutrient inadequacy among them. The most frequently consumed food group was cereals; meanwhile, the least was animal products. Their diets were energy-deficient and unbalanced with daily inadequate protein, vitamin A, and iron intake. The nutrition knowledge of the mothers/caregivers was poor as less than half of them had acceptable nutrition knowledge. Interventions to improve dietary practices and nutrient intake of the children such as nutrition education and supplementation should be intensified particularly among displaced families and their hosts in order to avert malnutrition and diet-related diseases among the children.

## Figures and Tables

**Table 1:** Demographic characteristics of the children.

Child's characteristics	Category	Frequency	Percentage (%)
Sex of child	Male	148	48.2
	Female	159	51.8
Age range of children	5-9years	125	40.7
	10-15years	182	59.3
Where the child lived before the crisis	Northwest	210	68.4
	Southwest	97	31.6
The year of child displacement	2017	80	26
	2018	88	28.7
	2019	88	28.7
	2020	37	12
	2021	14	4.5

**Table 2:** Demographic characteristics and socioeconomic status of the mothers/caregivers.

Parents/caregivers' characteristics	Category	Frequency	Percentage (%)
The highest academic level of parent/caregiver	No formal education	9	2.9
	Primary	107	34.9
	Secondary	161	52.4
	Higher	30	9.8

The occupation of parent/caregiver	Formally employed	25	8.1
	Self-employed	131	42.7
	Unemployed	151	49.2

Marital status of parent/caregiver	Married	202	65.8
	Divorced	17	5.5
	Never married	68	22.1
	Widow	20	6.5

Household income	Below 50,000	219	76
	50-150,000	65	21.2
	151-300,000	12	6.8
	Above 300,000	11	3.6

Number of people living in the house	Below 5	34	13.7
	5-8	155	50.4
	Above 8	118	35.9

Relationship of parent/caregiver to child	Mother	171	55.7
	Family relation	122	39.7
	Parent's friend	12	4
	Other	2	0.6

Age of mother/caregiver	Below 25	107	34.9
	25-50	175	57.0
	Above 50	25	8.1

Housing condition	Renting	212	69
	Living under another family.	81	26.4
	House owner	14	4.6

Main drinking water source	Good water source	234	76.2
	Bad water source	73	23.8

Toilet type	Pit toilet	248	80.8
	Flushing toilet	50	16.3
	No toilet	9	2.9

Religion	Christian	213	69.4
	Muslim	72	23.5
	Pagan	15	4.9
	Others	7	2.3

**Table 3:** Weekly frequency of consumption of various food groups.

Food groups	Total consumption	Frequency of consumption				
		0	1-2	3-4	5-6	>7
Cereals	97.7%	2.3%	8.8%	33.7%	34.2%	21%
Legumes and nuts	78.2%	2.9%	48.5%	22.3%	1.2%	6.0%
Roots and tubers	60.4%	35%	36.9%	17%	3.5%	7.6%
Animal products	42.3%	57.3%	21%	12.7%	6%	3%
Fruits	42.5%	57.5%	31%	18.3%	4.6%	3.6%
Vegetables	64.0%	40%	31.8%	13.1%	9.4%	5.7%
Fats, oil, and sugars	100%	0%	0%	0%	0%	100%

^∗^The percentages given under “total consumption” are the percentages of children who consumed each food group within seven days, and the figures under the “frequency of consumption” indicate the percentage of children that consumed a particular food group and the respective number of times each food group was eaten in a week.

**Table 4:** Proportion of different food groups consumed by the children within 24 hours prior to the survey.

Food groups	Frequency (*n*)	Percentage (%)
Cereals, white roots, tubers, and plantains	305	99.3
Beans and peas	96	31.3
Nuts and seeds	77	25.0
Diary	39	12.7
Meat, poultry, and fish	122	29.7
Eggs	21	6.8
Other vitamin A-rich vegetables and fruits	60	19.5
Dark green leafy vegetables	102	33.2
Other vegetables	224	73.0
Other fruits	28	9.1

**Table 5:** The nutrient intake of the children according to the 24-hour recall.

Nutrient	Mean daily intake	RDI for children aged 9-13years	Percentage (%) of children who meet their RDI
Energy	1640.5 ± 1.64 kcal	1745Kcal	61.2
Protein	18.45 ± 1.13 g	28 g	50.5
Retinol	470.27 ± 1.38 *μ*g	700 *μ*g	43
Iron	4.02 ± 0.08 mg	8 mg	32.2

**Table 6:** Level of nutrition knowledge on various aspects of nutrition.

Knowledge on specific aspects	*N*	%
Knowledge of good cooking practices	295	96.1
Aware of how to achieve good nutritional status	200	65.1
Aware of what a balanced diet is	224	72.9
Aware of protein-rich foods	72	23.5
Knowledge of the symptoms of marasmus	16	5.2
Knowledge of the cause of deficiency diseases	114	37.1
Knowledge of foods which are rich in iron	56	18.2
Aware of the consequences of iron deficiency	27	8.8
Aware of the consequences of vitamin A deficiency	143	46.6
Aware of vitamin A-rich foods	112	36.5

## Data Availability

The data (both quantitative and qualitative) used to support the findings of this study are available from the corresponding author upon request at nwachanmirabelle@gmail.com.
